# Sex disparities in STEMI care: pre-hospital delay and acute heart failure

**DOI:** 10.1186/s43044-026-00785-w

**Published:** 2026-09-22

**Authors:** Artur Dziewierz, Barbara Zdzierak, Tomasz Rakowski

**Affiliations:** 1 2nd Department of Cardiology, Jagiellonian University Medical College, Krakow, Poland; 2 Clinical Department of Cardiology and Cardiovascular Interventions, University Hospital in Krakow, Krakow, Poland

We commend Shaheen and colleagues for their important contribution to the literature on sex disparities in ST-segment elevation myocardial infarction (STEMI) [1]. Their analysis of the Egyptian cohort of the European Society of Cardiology STEMI Registry demonstrates several clinically important differences between women and men. Women were older, had a greater burden of cardiovascular comorbidities, experienced longer delays from symptom onset to first medical contact (FMC), and had nearly twice the in-hospital mortality of men (7.6% vs. 4.0%). These observations are consistent with previous studies demonstrating higher early mortality among women with STEMI across different populations [2–4].

One of the most clinically actionable findings is the longer pre-hospital delay among women. The time from symptom onset to FMC was 4.2 versus 3.1 h in women and men, respectively [1]. In contrast, once medical contact had occurred, FMC-to-primary PCI and FMC-to-thrombolysis times were similar between the sexes. Reperfusion strategies nevertheless differed: primary PCI was more frequent in women (53.6% vs. 48.1%), thrombolysis was less frequent (36.8% vs. 44.5%), and absence of reperfusion was slightly more common (9.6% vs. 7.4%) [1]. Thus, the findings highlight pre-hospital delay as a potentially important and modifiable contributor to the observed sex disparity, but they should not be interpreted as establishing a causal mechanism. This interpretation is consistent with previous work showing longer pre-hospital delays among women with STEMI and attenuation of the sex difference in 30-day mortality among patients presenting within 1 h of symptom onset [2].

Delay, however, is unlikely to provide the entire explanation. Women in the Egyptian registry were also more likely to experience advanced heart failure, with Killip class III–IV occurring in 12.8% of women compared with 6.9% of men [1]. Previous studies have similarly demonstrated that acute heart failure complicating STEMI is more frequent among women and is strongly associated with mortality [3, 4]. In a large international ACS cohort, women with STEMI had a higher risk of acute heart failure than men, while acute heart failure itself was associated with a several-fold increase in mortality [4]. These observations suggest that the greater clinical severity observed in women may constitute another important component of the mortality gap. However, the available registry data cannot determine whether delayed presentation, differences in myocardial vulnerability, comorbidity burden, or other unmeasured factors account for this association.

A particularly striking observation from the Egyptian registry is the very low use of emergency medical services (EMS). The emergency department represented the FMC for 83.2% of women and 81.0% of men, indicating that fewer than one in five patients entered the STEMI pathway through pre-hospital medical services [1]. This represents an important opportunity for improvement. Public education should emphasize recognition of the spectrum of ACS symptoms and immediate activation of EMS rather than self-transport to hospital. Importantly, EMS activation provides more than transportation: it permits pre-hospital diagnosis and ECG acquisition, appropriate triage and routing to reperfusion-capable centres, early treatment, and immediate management of life-threatening complications including cardiac arrest. Strategies aimed at increasing EMS use and shortening symptom-to-FMC time may therefore provide greater benefit than interventions focused exclusively on the in-hospital phase.

Longitudinal data from Poland indicate that sex differences in treatment delays and periprocedural mortality can narrow over time [5]. The Egyptian data similarly identify potentially modifiable targets, particularly patient delay and entry into organized emergency care. Future studies should directly investigate symptom presentation, reasons for delayed care seeking, EMS accessibility and utilization, socioeconomic determinants, and their contribution to sex-specific outcomes. Such data will be essential to move from documenting disparities to designing interventions capable of reducing them.

## Data Availability

No datasets were generated or analysed during the current study.

## Abbreviations

STEMI: ST-segment elevation myocardial infarction
FMC: First medical contact
PCI: Percutaneous coronary intervention
EMS: Emergency medical services
